# Methodology for Measuring Intraoperative Blood Loss: Protocol for a Scoping Review

**DOI:** 10.2196/58022

**Published:** 2024-10-16

**Authors:** Lätitia Dennin, Jörg Kleeff, Johannes Klose, Ulrich Ronellenfitsch, Artur Rebelo

**Affiliations:** 1 Department of Visceral, Vascular and Endocrine Surgery University Hospital of Halle (Saale) Martin-Luther-University Halle-Wittenberg Halle (Saale) Germany

**Keywords:** intraoperative blood loss, estimation of blood loss, intraoperative monitoring, surgery, surgical care, postoperative care, quality improvement

## Abstract

**Background:**

At present, there is no standardized method for measuring intraoperative blood loss. Rather, the current data on existing methods is very broad and opaque. In many cases, blood loss during surgery is estimated visually by the surgeon. However, it is known that this type of method is very prone to error. Therefore, better standardized methods are needed.

**Objective:**

This study aims to conduct a scoping review to present the currently available methods for measuring intraoperative blood loss. This should help to capture the current status and map and summarize the available evidence for measuring blood loss to identify any gaps.

**Methods:**

We will use a state-of-the-art methodological framework. The databases PubMed (MEDLINE) and Cochrane Library will be searched using a search strategy based on the PICO (Population, Intervention, Comparator, and Outcome) scheme. The search period will be limited to January 01, 2012, to December 31, 2023, and our search will be restricted to clinical trials or clinical studies, randomized controlled trials, and observational studies (in line with PubMed definition of study types). Only publications in English and German will be considered. The intention is to identify clinical studies that define “blood loss” as a target criterion or as a primary or secondary end point. EndNote (version 20.6; Clarivate) will be used for the screening process. The data will be collected and analyzed using Microsoft Excel (version 16.77.1).

**Results:**

The included studies will be listed in a database, and the following basic data will be extracted: title, year of publication, country, language, study type, surgical specialty, and type of procedure. The number of participants will be listed and the distribution of the participants will be documented in terms of gender and age. The following results are extracted: the type of measurement method used to measure blood loss in this study and whether the parameter “blood loss” was recorded as a primary or secondary outcome.

**Conclusions:**

Currently, there is no comparable review, resulting in ambiguous data regarding the prevailing measurement methods for intraoperative blood loss. The aim of this study is to provide a comprehensive overview—from methods of measurement to various formulae for calculating blood loss—and to establish a status quo. This could then serve as a foundation for further studies.

**International Registered Report Identifier (IRRID):**

DERR1-10.2196/58022

## Introduction

Every year, around 313 million operations are performed worldwide [1]. Intraoperative blood loss plays a very important role in the outcome of the patient in terms of perioperative morbidity and mortality [2]. There is currently no standardized method for recording, measuring, or estimating blood loss.

A common method, for example, is visual estimation using collection containers, abdominal drapes, and blood on the floor. The addition of irrigation fluid often leads to incorrect estimates of the amount lost, which can result in overestimates and underestimates by a factor of 2-3. Even longer professional experience did not provide any advantages in terms of a more accurate estimate [3]. Thus, the inaccuracies of the visual method and the consequences of misjudgment are well known. Nevertheless, visual estimation continues to persist due to the low effort and low costs involved [4].

To counteract this inaccuracy, newer methods have been developed, such as photometry [5]. However, this method is still largely unfamiliar to hospitals and medical staff [4]. Mathematical formulas for calculating blood loss are also available, some of which have been modified over the years or established from scratch [5,6]. Examples include the Gross equation [7], the Nadler formula [8], and the Meraculi equation [9]. For example, the Gross formula calculates blood loss by multiplying the patient’s blood volume by the initial hematocrit minus the minimum hematocrit divided by the average of the 2 latter values. The patient’s blood volume can either be estimated or determined using the Nadler formula. Meraculi’s formula also calculates the blood loss using the patient’s blood volume multiplied by the initial, respectively postoperative hematocrit. The first value is then subtracted from the latter. However, the formula takes into account transfusions, which are added. The reason is that the formula was initially described for a better transfusion strategy during operations.

Currently, the situation of the existing measurement methods and formulas has become very opaque, especially with regard to newly developed methods. However, the precise determination of blood loss forms the basis for improving surgical management and patient care, as well as the comparability of different surgeons, centers, surgical methods, and patient populations.

The aim of this scoping review is to provide an exploratory overview of the extent to which the methodology has been used in clinical studies (randomized controlled trials and others) with the end point or target criterion “intraoperative blood loss.” This is intended to record the current status and map the available evidence, but without going into the individual measurement methods in an evaluative manner. Our study, in contrast to those currently available, is intended to cover all specialties. This makes it possible to capture and classify the broad spectrum of surgery and surgical procedures. It summarizes the available evidence base for measuring blood loss in order to identify any gaps and can serve as a starting point for further studies.

## Methods

The scoping review will follow the methodological framework published by Arksey and O’Malley [10] and the further development and refinement of the methods by Levac et al [11]. This framework goes a long way to ensuring the quality of scoping reviews and promoting robustness and validity. For reporting, the PRISMA-ScR (Preferred Reporting Items for Systematic Reviews and Meta-Analyses extension for Scoping Reviews) guidelines will be used [12].

The primary purpose of a scoping review is to explore a complex and multilayered topic that has not yet been comprehensively investigated and, if necessary, to fundamentally categorize it in order to gain an initial overview as representative as possible of the data situation. Therefore, the intention is not to cover the entire available literature but predominantly to create a comprehensive foundation sample. In this context, the research question is designed to be as open as possible and the identification of relevant results will be determined iteratively. This is based on the fact that with increasing familiarity with the data situation, better knowledge of the unexplored topic area is gained, and the search strategy should be reflected upon and critically adapted to the newly acquired knowledge as necessary [10]. The process guarantees, on the one hand, that the subject area is comprehensively presented or categorized for the first time and, on the other hand, that the boundaries of the research environment are clearly articulated [11].

PubMed was chosen as the primary database. The search is also extended to the Cochrane Library database. This is another international library that comprises 3 scientific databases: the Cochrane Database of Systematic Reviews (CDSR, Cochrane Reviews), Cochrane Clinical Answers (CCAs), and Cochrane Central Register of Controlled Trials (CENTRAL, Trials). In our scoping review, we will rely on the latter, and all trials will be included in it, even those from the various original sources of publication (MEDLINE through PubMed, Embase, CINAHL, ClinicalTrials.gov, and ICTRP). We will not use a third database, as a very large number of studies could already be found in an initial search. We justify this by stating that we cover the 2 most important databases and that a systematized search that includes all possible literature is not the main objective of the scoping review.

We have decided to limit the time period to the last 11 years, as the topic of methods for measuring intraoperative blood loss is very diverse and complex, and there is a large number of existing studies. In this way, our scoping review will be able to cover the methods currently in use and answer the key questions on the data situation in order to initiate more specific studies if necessary. Therefore, studies published from January 01, 2012 to December 31, 2023, will be included.

We will only include studies in English or German because English is the standard language in research and thus encompasses the majority of studies, and German is spoken by all authors of this scoping review.

Our search will be limited to clinical trials or clinical studies, randomized controlled trials, and observational studies (in line with PubMed’s definition of study types). This restricts our scope to primary research and excludes secondary research such as reviews. In addition, case reports, commentaries, or letters will be excluded, as these only meet the research standards to a limited extent.

Only studies that include humans as study participants will be considered. Therefore, studies in which experiments were conducted on animals will be excluded.

### Search Strategy

In order to pursue the research question, a search strategy was developed using the PICO scheme (Population, Intervention, Comparator, and Outcome). We will proceed as follows.

First, the databases will be studied using this standardized search. Multimedia Appendix 1 shows the search strategy that will be used in PubMed (MEDLINE). The same search strategy will be used for the Cochrane Library, which can be found in Multimedia Appendix 2.

Second, the bibliographies of the included studies will be manually searched for further suitable studies. The abstracts will be read independently by 2 authors and will be evaluated with regard to the inclusion and exclusion criteria. Differences of opinion between the authors will be settled by mutual agreement. If no agreement can be reached, a third reviewer will evaluate the study and decide on inclusion or exclusion. The decision-making process in the literature search and the selection of studies will be supplemented by a flow chart in the final report (more details in Multimedia Appendix 3). During the selection process, the studies will be extracted separately by the 2 authors and will be collected in a separate database.

For the screening process, the software Endnote (version 20.6; Clarivate) will be used.

The inclusion and exclusion criteria are summarized in Table 1.

**Table 1:** Inclusion and exclusion criteria.

	Inclusion criteria	Exclusion criteria
Database	PubMed Cochrane Library	—^a^
Study type	Randomized controlled trial Clinical study, Clinical trial Observational study	Reviews Case reports Case series with less than 5 patients Commentaries Letters
Study population	Humans	Animals
Reported outcomes	Primary outcome: Measurement method for intraoperative blood loss Secondary outcomes: Measurement method unknown (study that measures blood loss but does not specify a method) “Blood loss” as a primary or secondary outcome	—
Language	English German	Other language

^a^Not available.

### Data Extraction

The included studies will be listed in a database using Microsoft Excel (version 16.77.1), and the following basic data will be extracted: Title, year of publication, country, and language. The number of participants will be listed, and the distribution of the participants will be documented in terms of gender and age. For better evaluation and comparability, we will divide the age into groups: infants (up to the age of 3 years), children (ages 4-12 years), adolescents (ages 13-18 years), young adults (ages 19-30 years), adults (ages 31-60 years), older adults aged 61-80 years, and older adults aged ≥81 years.

Finally, the following data will be extracted: study type, surgical specialty, type of surgery, measurement method, and “blood loss” as a primary or secondary outcome.

A preselection will be made for the respective points provided in Textbox 1.

In the event that a study does not differentiate outcomes directly into primary and secondary end points, the outcomes are all considered “primary” and will be included in our scoping review, too.

**Preselection criteria.**

- Study type:
  Intervention study
    - Randomized controlled trial
    - Controlled clinical trial
  Observational study (case-control study and cohort study)
    - Prospective
    - Retrospective
- Surgical specialty:
  Vascular surgery
  Trauma surgery and orthopedics
  Thoracic surgery
  Visceral surgery
  Plastic surgery
  Pediatric surgery
  Cardiac surgery
  Ear, nose, and throat surgery
  Neurosurgery
  Gynecology
  Urology
  Oral and maxillofacial surgery
  Eye surgery
  Endocrine surgery
  Spinal surgery
  Multidisciplinary interventions
  Emergency general surgery
- Type of procedure: Name of the operation, eg “Appendectomy.”
- Measurement method:
  Visually estimated or bleeding scores (specify the score)
  Measured using measurement data (specify the method used)
  Calculated using a formula (specify the formula used)
  Unknown
- Blood loss as an outcome or parameter:
  Primary outcome
  Secondary outcome
  Not defined as an outcome, but one of the target criteria of the study

## Results

The results will be collected in a database and will be presented in tabular form or diagrams using Microsoft Excel (version 16.77.1). For a better overview, the data will be grouped according to the type of method (measurement methods, estimation methods, and calculation methods). Reviewing the studies has been started. Evaluation and analysis of the data is planned to be finished in 2025.

## Discussion

The data summary facilitates the comparison of evaluation methods and the identification of dependencies. It allows for an assessment of the methods in terms of their precision and required effort. In addition, it will help to uncover patterns, such as the prevalence of certain methods in various surgical specialties. Questions can be explored, like whether there is a link between the complexity or accuracy of a method and the time of publication or the quality of the study (eg, randomized vs observational studies). These aspects, among others, present opportunities for comparison.

Up to now, there has been no comparable review. An existing study has investigated methods for quantifying blood loss in orthopedic trauma [13]. In the field of obstetrics, 3 studies have been published on estimating blood loss: 2 literature reviews [14,15] and 1 systematic review [16]. There is also a systematic review highlighting the strengths and weaknesses of currently used measurement methods [5], which only included studies that examined the accuracy of blood loss quantification techniques in vivo and in vitro.

Currently, the measurement of intraoperative blood loss lacks clarity due to the absence of standardized methods. A variety of methods are used in practice, often with personal modifications. This inconsistency extends to the formulas used for calculating blood loss. Such variability could pose challenges for our scoping review, particularly in categorizing the methods used in different studies. In addition, there is a potential for bias if multiple studies fail to detail their measurement methods. To mitigate this issue, we plan to include these studies in our collection and consider this factor in our analysis.

Overall, this approach offers the opportunity to identify existing gaps and propose ideas for standardization.

## Appendix

Multimedia Appendix 1 PubMed search strategy.

Multimedia Appendix 2 Search strategy Cochrane Library.

Multimedia Appendix 3 PRISMA (Preferred Reporting Items for Systematic Reviews and Meta-Analyses) 2020 flow diagram.

## Abbreviations

CCAs: Cochrane Clinical Answers
CDSR: Cochrane Database of Systematic Reviews
PICO: Population, Intervention, Comparator, and Outcome
PRISMA-ScR: Preferred Reporting Items for Systematic Reviews and Meta-Analyses extension for Scoping Reviews

## Data Availability

The datasets generated during and/or analyzed during this study are available from the corresponding author upon reasonable request.
