# Supplementary material for: Methodology for Measuring Intraoperative Blood Loss: Protocol for a Scoping Review
Source: JMIR Res Protoc. 2024 Oct 16;13:e58022. doi: 10.2196/58022 (PMC11525073; doi:10.2196/58022)
Supplement: Multimedia Appendix 1 [file resprot_v13i1e58022_app1.docx]

| Search strategy PubMed | |
| --- | --- |
| **P** | ("intraoperat*"[Title/Abstract] OR "intra-operat*"[Title/Abstract] OR “intraop*[Title/Abstract])  AND  ("surgery"[MeSH Subheading] OR "surgery"[Title/Abstract] OR "surgical procedures, operative"[MeSH Terms] OR "operative surgical procedures"[Title/Abstract] OR "general surgery"[MeSH Terms] OR "general surgery"[Title/Abstract] OR "operation"[Title/Abstract]) |
| **I** | ("measurement"[Title/Abstract] OR "measure"[Title/Abstract] OR "estimate"[Title/Abstract] OR "estimating"[Title/Abstract] OR "calculat*"[Title/Abstract] OR "photometr*"[Title/Abstract] OR "weights and measures"[MeSH Terms] OR "weights"[Title/Abstract] OR "monitoring, intraoperative"[MeSH Terms] OR "equation*"[Title/Abstract] OR "formula"[Title/Abstract] OR "gross equation"[Title/Abstract] OR "Nadler"[Title/Abstract] OR "hematocrit*"[Title/Abstract] OR “meraculi”) |
| **C** |  |
| **O** | ("hemorrhage"[MeSH Terms] OR "hemorrhage"[Title/Abstract] OR "blood loss, surgical"[MeSH Terms] OR "blood loss"[Title/Abstract] OR "intraoperative complications"[MeSH Terms] OR "bleed*"[Title/Abstract]) |

### **P** AND **I** AND **O**
