# Supplementary material for: Methodology for Measuring Intraoperative Blood Loss: Protocol for a Scoping Review
Source: JMIR Res Protoc. 2024 Oct 16;13:e58022. doi: 10.2196/58022 (PMC11525073; doi:10.2196/58022)
Supplement: Multimedia Appendix 2 [file resprot_v13i1e58022_app2.docx]

| search strategy Cochrane Libary | |
| --- | --- |
| #1 | (intraoperat*):ti,ab |
| #2 | (Intraop*):ti,ab |
| #3 | {or #1-#2} |
| #4 | MeSH descriptor: [General Surgery] explode all trees |
| #5 | (surgery):ti,ab |
| #6 | MeSH descriptor: [Surgical Procedures, Operative] explode all trees |
| #7 | (operative surgical procedures):ti,ab |
| #8 | MeSH descriptor: [General Surgery] explode all trees |
| #9 | (general surgery):ti,ab |
| #10 | (operation):ti,ab |
| #11 | {or #4-#10} |
| #12 | (measurement):ti,ab |
| #13 | (measure):ti,ab |
| #14 | (estimate):ti,ab |
| #15 | (estimating):ti,ab |
| #16 | (calculat*):ti,ab |
| #17 | (photometr*):ti,ab |
| #18 | MeSH descriptor: [Weights and Measures] explode all trees |
| #19 | (weights):ti,ab |
| #20 | MeSH descriptor: [Monitoring, Intraoperative] explode all trees |
| #21 | (equation*):ti,ab |
| #22 | (formula):ti,ab |
| #23 | (gross equation):ti,ab |
| #24 | (nadler):ti,ab |
| #25 | (hematocrit*):ti,ab |
| #26 | {or #12-#25} |
| #27 | MeSH descriptor: [Hemorrhage] explode all trees |
| #28 | (hemorrhage):ti,ab |
| #29 | MeSH descriptor: [Blood Loss, Surgical] explode all trees |
| #30 | (blood loss):ti,ab |
| #31 | MeSH descriptor: [Intraoperative Complications] explode all trees |
| #32 | (bleed*):ti,ab |
| #33 | {or #27-#32} |
