# Supplementary material for: Methodology for Measuring Intraoperative Blood Loss: Protocol for a Scoping Review
Source: JMIR Res Protoc. 2024 Oct 16;13:e58022. doi: 10.2196/58022 (PMC11525073; doi:10.2196/58022)
Supplement: Multimedia Appendix 3 [file resprot_v13i1e58022_app3.docx]

**Identification of studies via databases**

Records removed *before screening*:

Duplicate records removed (n = )

Records removed for other reasons (n = )

Records identified from:

Databases (n = )

**Identification**

Records screened (via Abstract)

(n = )

Records excluded

(n = )

Reports sought for retrieval

(n = )

Reports not retrieved

(n = )

**Screening**

Reports assessed for eligibility

(n = )

Reports excluded:

Reason 1 (n = )

Reason 2 (n = )

Reason 3 (n = )

etc.

Studies included in review

(n = )

Reports of included studies

(n = )

**Included**
